# Use of simulation to optimize a sweet corn breeding program: implementing genomic selection and doubled haploid technology

**DOI:** 10.1093/g3journal/jkae128

**Published:** 2024-06-13

**Authors:** Marco Antônio Peixoto, Igor Ferreira Coelho, Kristen A Leach, Thomas Lübberstedt, Leonardo Lopes Bhering, Márcio F R Resende

**Affiliations:** Laboratório de Biometria, Universidade Federal de Viçosa, Viçosa, Minas Gerais 36570-900, Brazil; Sweet Corn Breeding and Genomics Lab, University of Florida, Gainesville, FL 32611, USA; Laboratório de Biometria, Universidade Federal de Viçosa, Viçosa, Minas Gerais 36570-900, Brazil; Sweet Corn Breeding and Genomics Lab, University of Florida, Gainesville, FL 32611, USA; Sweet Corn Breeding and Genomics Lab, University of Florida, Gainesville, FL 32611, USA; Department of Agronomy, Iowa State University, Ames, IA 50011, USA; Laboratório de Biometria, Universidade Federal de Viçosa, Viçosa, Minas Gerais 36570-900, Brazil; Sweet Corn Breeding and Genomics Lab, University of Florida, Gainesville, FL 32611, USA

**Keywords:** breeding program design, breeding analytics, hybrid performance, stochastic simulation, vegetable breeding

## Abstract

Genomic selection and doubled haploids hold significant potential to enhance genetic gains and shorten breeding cycles across various crops. Here, we utilized stochastic simulations to investigate the best strategies for optimize a sweet corn breeding program. We assessed the effects of incorporating varying proportions of old and new parents into the crossing block (3:1, 1:1, 1:3, and 0:1 ratio, representing different degrees of parental substitution), as well as the implementation of genomic selection in two distinct pipelines: one calibrated using the phenotypes of testcross parents (*GSTC* scenario) and another using F_1_ individuals (*GSF1*). Additionally, we examined scenarios with doubled haploids, both with (*DH*) and without (*DHGS*) genomic selection. Across 20 years of simulated breeding, we evaluated scenarios considering traits with varying heritabilities, the presence or absence of genotype-by-environment effects, and two program sizes (50 vs 200 crosses per generation). We also assessed parameters such as parental genetic mean, average genetic variance, hybrid mean, and implementation costs for each scenario. Results indicated that within a conventional selection program, a 1:3 parental substitution ratio (replacing 75% of parents each generation with new lines) yielded the highest performance. Furthermore, the *GSTC* model outperformed the *GSF1* model in enhancing genetic gain. The *DHGS* model emerged as the most effective, reducing cycle time from 5 to 4 years and enhancing hybrid gains despite increased costs. In conclusion, our findings strongly advocate for the integration of genomic selection and doubled haploids into sweet corn breeding programs, offering accelerated genetic gains and efficiency improvements.

## Introduction

Genomic selection is a well-established technology and an integral part of modern plant breeding programs. Its effectiveness in augmenting genetic gain is widely acknowledged, with numerous instances where it outperforms traditional selection methods (Bančič *et al*. 2021; Peixoto *et al*. 2023; Werner *et al*. 2023). The superiority of genomic selection over traditional approaches, such as phenotyping and best linear unbiased prediction (BLUP) estimates, can be attributed to several key factors. First, genomic selection enhances the precision of selection, increasing the confidence in selecting genotypes throughout successive breeding cycles, something that is invaluable for low-heritability traits. The increased certainty in genotype selection can expedite the breeding cycle by reducing the generation interval, especially when combined with strategies like rapid cycle implementation (de Jong *et al*. 2023; Peixoto *et al*. 2023). Moreover, genomic selection offers a feasible solution for evaluating traits that are challenging to measure or are associated with inherent difficulties (Calus *et al*. 2013). By capturing the linkage disequilibrium between molecular markers and the trait of interest via QTLs within the training population, it is also possible to predict the performance of non-phenotyped plants in the target population.

To successfully leverage genomic selection and integrate it into breeding programs, breeding pipelines have undergone significant transformations (Gaynor *et al*. 2017; Powell *et al*. 2020; Merrick *et al*. 2022). Nowadays, it stands as a vital tool for maximizing genetic gain, particularly in row crops such as field corn. The breeding processes of sweet and field corn share many similarities such as the development of inbreds, production of hybrids, and overlapping target traits. However, sweet corn lacks the well-defined heterotic groups of field corn (Zystro *et al*. 2021a; Peixoto *et al*. 2023). A possible explanation for this is the larger number of key traits needed to make a successful sweet corn hybrid, besides yield. As a specialty vegetable crop, table quality, *i*.*e*. flavor, texture, and ear appearance, is an important factor in sweet corn evaluation. Many qualitative traits have recessive or additive inheritance, meaning both parents must contribute the same allele(s) to the hybrid. As a result, these parents may have been developed from parental lines derived from the same high-quality inbred (Tracy 2001). These unique challenges must be kept in mind when considering sweet corn improvement.

There are several tools currently utilized in field corn that could better optimize sweet corn breeding, including parental selection optimization, the implementation of genomic selection for line development, and the creation of doubled haploid lines. Recently, studies have explored different tools to optimize breeding programs through simulation and comparison of breeding scenarios with stochastic processes. Simulations have been widely adopted in different crops, such as wheat (Tessema *et al*. 2020), rice (Sabadin *et al*. 2022), soybean (Silva, Xavier, *et al*. 2021), field corn (Cowling *et al*. 2020), and sweet corn (Peixoto *et al*. 2023). Simulations have the potential to quickly evaluate and develop many different strategies and conditions for inclusion into a plant breeding program before committing to cost-inhibitive field experimentation (Faux *et al*. 2016).

Genome selection applied to hybrid crops can be useful in breeding programs through the building of predictive models. These models may (i) increase accuracy by adopting an estimated breeding value to select the best genotypes, and (ii) by reducing the genotype selection interval (Jannink *et al*. 2010; Heffner *et al*. 2011; Fritsche-Neto *et al*. 2021). Doubled haploid technology reduces the time to generate highly homozygous lines compared with successive generations of selfing individuals. In hybrid crops such as maize, the combination of both genomic selection and doubled haploids has been highly recommended because of its positive impact on the breeding program in terms of genetic gain and cycle time reduction (Andorf *et al*. 2019; Boerman *et al*. 2020).

This study was designed to investigate the implementation of genomic selection and doubled haploids into the current sweet corn breeding pipeline at the University of Florida (UF) using simulated data. The aim was to optimize genetic gain using tools that were both technically and economically feasible to integrate into the program. Our objectives were to determine: (i) the rate of parental substitution that resulted in the greatest genetic gain, (ii) the optimum training set population with which to train a genomic selection model, (iii) the impact of doubled haploids on genetic gain, and (iv) the cost of implementing such strategies in the program.

## Material and methods

Stochastic simulations were adopted to mimic a real breeding program, based on the current sweet corn breeding program pipeline from the UF. We compared the genetic mean and genetic variance over a window of 20 years of breeding for three core breeding scenarios: conventional truncated selection, genomic selection, and doubled haploid integration. First, we describe the stochastic approach used to implement these scenarios in the sweet corn breeding program. Then, we define the various breeding scenarios, including the conventional breeding program, varying ratios of parental substitution, the implementation of genomic selection, and doubled haploids, along with various combinations of each of these. Finally, we describe the methods used to evaluate and compare these scenarios.

### Stochastic simulations of the sweet corn breeding program

A base genome consisting of 100 individuals in Hardy–Weiberg equilibrium was simulated to represent the genetic variation present in sweet corn using the Markovian Coalescent Simulator (Chen *et al*. 2009), available in AlphaSimR (Gaynor *et al*. 2021; R Development Core Team 2022). The option “*Maize*” in the argument “*species*” was set for haplotype creation, generating a total of 10 chromosome pairs, where each chromosome pair has a genetic length of 2.0 Morgans and a physical length of 2 × 108 base pairs. A recombination rate of 1.25 × 10^−8^ and mutation rate of 2.5 × 10^−8^ was also included within the option *Maize* (Hickey *et al*. 2014; Powell *et al*. 2020). We then generated 300 evenly distributed quantitative trait nucleotides per pair of chromosomes. To build a based genome that mimicked the historic public–private split reported for sweet corn genotypes in the United States (Tracy 1993), two different populations were split and let evolved independently for 30 generations. A summary of the simulations’ phases and parameters is provided in Supplementary Table 1.

A quantitative trait with additive, dominance, and genotype-by-environment (GE) effects was simulated. The additive genetic mean and variance of the simulated trait were set to 150 and 30, respectively. The dominance effect was calculated according to the trait's degree of dominance, with a mean of 0.93 and a variance of 0.3, roughly following the historical levels of heterosis in field corn (Troyer and Wellin 2009; Powell *et al*. 2020). For the implementation of genomic selection, 3,000 single nucleotide polymorphisms (SNPs) were randomly allocated across each chromosome pair, for a total of 30,000 SNPs per genome.

The GE calculation included the mean and the variance of the target trait and included an environmental component (EC) with a mean of 0 and a variance of 1. Both, the trait mean and variance were included as specific terms and scaled to achieve the founder population mean and variance specified by the user. The EC was set with two different magnitudes in this study. To replicate the GE interaction, present in the sweet corn breeding program at the UF, a target environment was used for population advancement and hybrid assessment while an off-season environment was used for population advancement and crossing purposes. To represent these two dissimilar environments, we adopted EC variables with different intensities. The first EC accounted for the genotype-by-year effect (EC_gy_), representing the evaluation of genotypes that were planted in a single target environment in different years. The EC_gy_ helps to account for weather differences between years at a single location. In this case, values for the EC_gy_ were sampled from a normal distribution with a mean equal to 0.5 and a standard deviation of 0.03. The second EC accounts for the genotype-by-year-by-environment effect (EC_gye_), representing a different environment (off-season environment) planted in different seasons and different years. The EC_gye_ accounts for weather differences across years, as well as variation in soil, altitude, and weather between locations. Here, EC_gye_ values were sampled from a normal distribution with a mean equal to 0.1 or 0.9 (by chance) and a standard deviation of 0.03.

Two different values of GE interaction variances were evaluated in this study. The values were related to the additive variance, being 0 and 10 times the scaled additive variance (the additive variance was 30 in the simulated traits). Furthermore, to represent two independent traits, we simulated one with a low broad sense heritability (*i*.*e*. a complex trait, controlled by many genes with small effects, and highly influenced by the environment) and a another with high broad sense heritability (*i*.*e*. a complex trait controlled by a few genes and slightly influenced by the environment).

Throughout the pipeline, phenotypes were generated by adding random error to the genetic values of the target trait. The random error was sampled from a normal distribution with a mean of zero and an error variance of $\sigma_{res}^{2}$. The error variance was then defined by the target trait heritability at each testing stage. Entry-mean narrow sense heritability followed the number of replicates in each phase of the breeding program and increased throughout the pipeline once the number of replicates increased. In addition, the simulations were implemented independently for two distinct trait heritabilities, combining those levels of residual variance: 60 and 240 (high- and low-heritability, respectively).

### Breeding scenarios

The current sweet corn breeding program at UF uses reciprocal recurrent selection from two pseudo-heterotic groups, one with UF lines and another from commercially released lines (where the tester comes from), to predict the general combining ability (GCA) performance of UF material (parents) via testcross evaluations. To generate the testers (commercial lines) a private breeding program was developed/simulated in parallel with the main program, with phenotypic-based truncated selection applied. The best lines at the end of each cycle (five lines) were selected to be the testers and used to be the testcross parents (mimicking elite private inbreds).

**Fig. 1.**
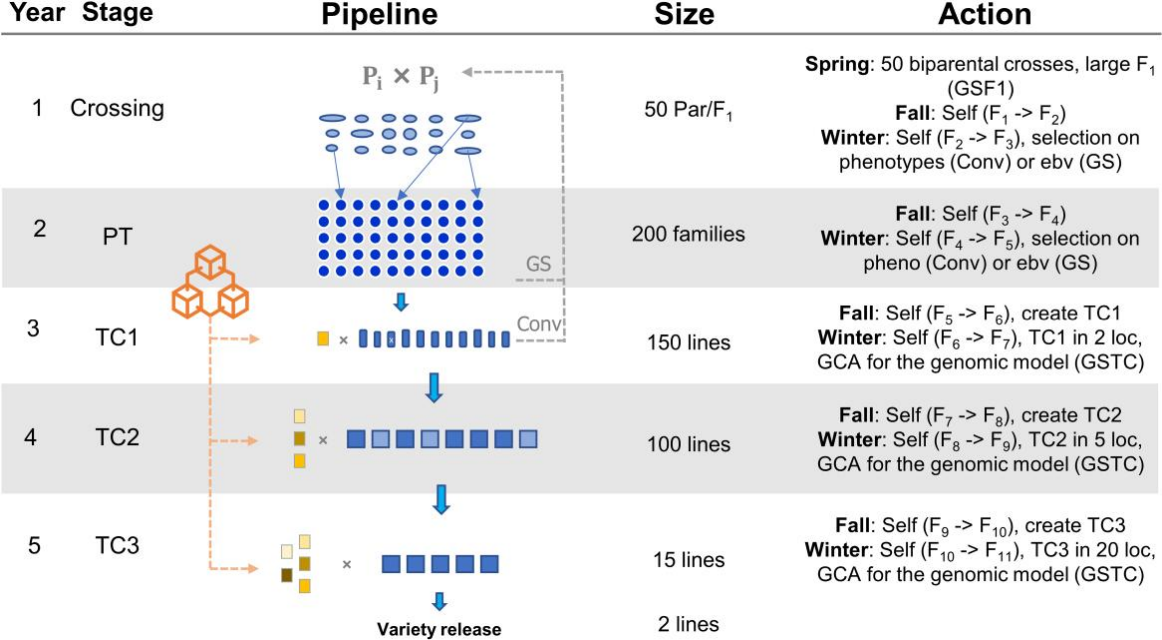
Sweet corn breeding program pipeline. A cycle in this pipeline is completed in 5 years to generate two new elite lines. Individuals were evaluated based on observed phenotype and estimated breeding values. Testcross 1 (TC1) is evaluated in two environments, testcross 2 (TC2) in five environments, and testcross 3 (TC3) in 20 different environments, using respectively one, three, and five testers to generate the hybrids that were assessed at the target environment. PT, preliminary trials; *Conv*, conventional breeding program; GCA, general combining ability; *GCF1*, genomic selection with F_1_ training population; *GSTC*, genomic selection with testcross training population. Dotted lines on the right of the Pipeline column with GS/Conv mean the stage where the individuals are selected for the crossing block. Dotted lines on the left of the Pipeline column with the features represent the private program that generates inbreed lines used as testers against the top lines from the main program.

For the conventional breeding program, we simulated a 15-years burn-in phase using the conventional breeding pipeline (*Conv*) to represent the cycles of selection (current pipeline of the sweet corn breeding program; Fig. 1). Then, 50 elite parents were crossed to generate 50 new F_1_s. These F_1_s were successively self-pollinated and selected based on their observed phenotypic values (truncated selection), resulting in inbreds. These inbreds were evaluated for hybrid performance in three rounds of testcrosses (TC1–TC3) against elite private inbreds (being developed in parallel) and represented hybrid tests in the target environment (winter season). Each level of TC differed in the number of testers with which individuals were crossed and the number of trials in which they were evaluated. TC1 consisted of one tester and two trials, TC2, three testers and five trials, and TC3, five testers and 20 trials. From each testcross, the top-performing individuals were selected to progress to the next stage. From TC3, the top two inbred lines were selected for commercial release. The overall result of the burn-in period was a population whose genetic mean and variance, and allelic frequencies mimicked a population currently under selection. This provided a consistent starting point from which every scenario could begin.

Since private breeding programs are notably larger than conventional programs, the parallel private breeding program mirrored the conventional program except that each stage described above was set to be four times as large, with five elite inbreds slated for commercial release.

The current breeding program being employed at the UF (*Conv* scenario as previously described) was used as the breeding program of the burn-in phase and as a reference pipeline. Thus, all the strategies implemented in this study were compared to the benchmark *Conv* scenario, which represents the real, or at least very close to the real number of plots, program costs (*e*.*g*. land use and labor), and the cycle of the sweet corn breeding program from the UF (Fig. 1).

The first objective of this study was to determine the degree of parental substitution for the individuals selected for the crossing block to randomly mate and generate the F_1_ population. To address this, four distinct scenarios of recurrent selection cycles were run, each utilizing a different ratio of old parents to new lines, those being 1:3, 1:1, 3:1, 0:1. For the discrete scenario (0:1), parents were only selected from the currently breeding cycle, while for the overlapping strategies (1:3, 1:1, and 3:1, the numbers refer to the relative proportions of old and new individuals used as parents, respectively), the new lines were selected from the top 50 lines of the F_5_ populations ranked by GCA (*Conv* scenario) and combined with lines that were parents in the previous cycle only.

The second objective was to optimize the training population used to develop and implement a genomic selection model. Two types of training sets were chosen for being feasible to implement in the current UF sweet corn breeding pipeline. The first scenario, *GSF1*, used 800 F_1_ families as the training population. The second scenario, *GSTC*, used parents from the UF pseudo-heterotic group side that were evaluated in all three testcrosses (TC1–TC3) as the training population. In each scenario, observed phenotypic values for the trait of interest within the target environment were assessed. After training each model with their respective training populations, the models were used to predict breeding values for individuals in the F_3_ and F_5_ generations. For both GS scenarios, the individuals selected to compose the crossing block came from the F_5_ generation, which were ranked by breeding values, and the top lines selected (Fig. 1).

The four parental substitution ratios described above were again implemented in both genomic selection scenarios. This resulted in 12 total scenarios, where each of the three main pipelines, *Conv*, *GSTC*, and *GSF1*, were run with four different degrees of parental substitution. From these 12 scenarios, the top-performing parental substitution ratio and the top-performing genomic selection training population model were selected for later use.

**Fig. 2.**
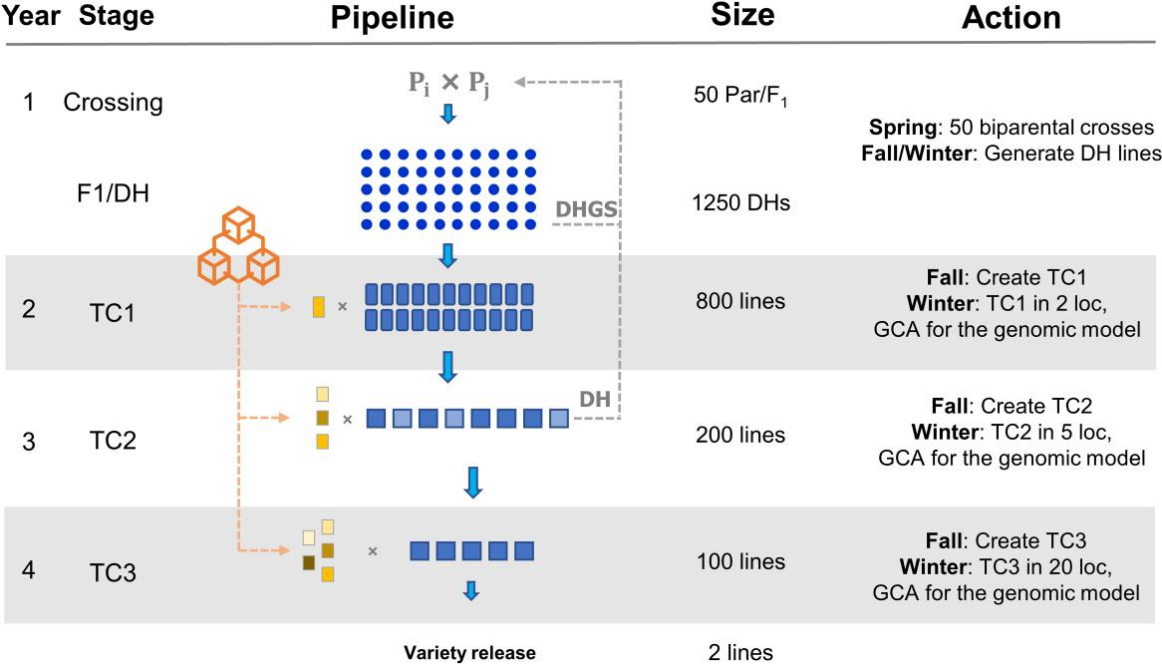
Sweet corn doubled haploid breeding program. One breeding cycle consists of 4 years and generates two new elite lines. Testcross 1 (TC1) is evaluated in two environments, testcross 2 (TC2) in five environments, and testcross 3 (TC3) in 20 different environments, using, respectively, one, three, and five testers to generate the hybrids that were assessed at the target environment. Evaluation of individuals was based on phenotypic values (phenotypes) and estimated breeding values. *DH*, doubled haploid with phenotypic selection; *DHGS*, doubled haploid with genomic selection; GCA, general combining ability. Dotted lines on the right of the Pipeline column with DH/DHGS represent the stage where the parents were selected based on phenotypes (*DH* scenario) and estimated breeding values (*DHGS* scenario). Dotted lines on the left of the Pipeline column with the features represent the private program that generates inbreed lines used as testers against the top lines from the main program.

The third objective was to assess the integration of doubled haploids into the breeding pipeline. To this end, two new implementations were assessed. The first looked at the integration of doubled haploids into a conventional program (*DH* scenario) and the second looked at the joint integration of doubled haploids and genomic selection (*DHGS* scenario). Doubled haploid technology shortens the breeding cycle by producing genetically homozygous lines in two generations. Thus, in both scenarios, doubled haploids were deployed in the first year of a conventional pipeline after new crosses had been generated, shortening the total breeding cycle to 4 years (Fig. 2). In the *DH* scenario, all the selections were made based on the phenotypic means of the genotypes. The GCA was estimated for each individual, and a mean was taken from among the values from different testers. Those with higher phenotypic value progressed to the next year, while the parents (using the 3:1 strategy) came from the top-ranked individuals in the second testcross (TC2). In the *DHGS* scenario, individuals were selected based on breeding values predicted by the genomic selection model trained as in the *GSTC* scenario described above. Selected hybrids were planted and the parental GCA values were estimated. These values were used to compose the calibration set of the next cycle, while the genomic model previously described was used to predict the individual's performance. The best-performing individuals were selected for the next cycle based on estimated breeding values while the top recently created doubled haploids (year 1) were selected to compose the crossing block (early selection).

To evaluate the importance of the breeding program size in the parameters and scenarios proposed here, we implemented a new set of simulations where a larger breeding program was used, about four times the size of the one before mentioned (hereafter 200 crosses scenario).

### Breeding value estimation via genomic models

Marker effects were estimated by fitting a random regression best linear unbiased prediction (RRBLUP) model (Meuwissen *et al*. 2001). Genomic predictions were made using the RRBLUP function implemented in AlphaSimR (Gaynor *et al*. 2021), which uses the efficiently mixed-model association (EMMA) algorithm to estimate variance components using restricted maximum likelihood (REML) and returns marker effects. We fed the model with a training population containing both genotypic and phenotypic information for each individual. They were then used to estimate breeding values for the individuals in the target population, made available only by markers.

The training population was updated using a 4-year sliding window approach, using the most recent 4 years of genotypic and phenotypic data from the training population. In the *GSF1* scenario, a population of 800 F_1_ families was created and assessed in the target environment. Then the genotypes and phenotypes were used to calibrate the genomic selection model. For the *GSTC* scenario, the training population consisted of parents from the UF pseudo-heterotic group of the conventional breeding program for each testcross (150, 100, and 15 individuals for TC1, TC2, and TC3, respectively). These parents’ performance, assessed by GCA, together with marker information, were fed to the model. In the *DHGS* scenario, a similar strategy was used but with larger testcross populations (800, 200, and 100 individuals for TC1, TC2, and TC3, respectively). For the GCA model where more than one tester was used (three testers for TC2, and five testers for TC3), the GCAs were regressed into the individuals, and means were recovered for each parent originating from the UF pseudo-heterotic group.

### Comparison of breeding programs

All scenarios were run in 50 repetitions and every breeding scenario was compared using the genetic mean and the average genetic variance across those repetitions. These parameters were estimated for the parents at the beginning of each cycle. In addition, we estimated the hybrid performance in the second set of simulations by calculating the genetic mean of the TC3 hybrids. The costs to implement each scenario were defined in terms of field plots, genomic selection samples for genotyping, phenotypic assessment, and doubled haploid production.

To estimate the monetary costs associated with each scenario, the following base costs were assumed: $12 for an inbred advancement field plot, $8 for a testcross hybrid field plot, $10 to genotype each sample, $4 to phenotype each plot, and $20 to produce a single doubled haploid line from an F_1_ family. Annual costs were calculated as the sum of the costs per plot, the costs to genotype the samples, and the cost to produce doubled haploids (Table 1). Estimates of relative genetic gain (relative gain) were made by assuming the genetic gain of the *Conv* scenario as a unit (1) and scaling the other scenarios’ genetic gain concerning the *Conv* scenario. The cost efficiency was measured by dividing the relative gain by the relative costs (assuming the costs of the *Conv* scenario as a unit and scaling the other scenarios’ costs to that one). It gives a measure of how much cost by a unit of genetic gain is needed in each scenario.

**Table 1.** Summary of the costs and number of plots for each year into the breeding program pipeline of the simulated scenarios for the sweet corn breeding program of the University of Florida.

Year	*Conv*		*GSTC*		*GSF1*		*DH*		*DHGS*	
	Plots	Cost ($)	Plots	Cost ($)	Plots	Cost ($)	Plots	Cost ($)	Plots	Cost ($)
*50 Crosses*										
1	200	2,800	200	2,800	3,400	62,000	50	25,600	50	25,600
2	600	8,800	600	11,800	600	11,800	1,829	28,348	1,829	40,848
3	1,320	17,040	1,320	29,040	1,320	29,040	960	12,720	960	12,720
4	960	12,320	960	13,320	960	12,320	520	6,400	520	6,400
5	475	5,820	475	5,970	475	5,820	—	—	—	—
Total	3,555	46,780	3555	62,930	6,755	120,980	3,359	73,068	3,359	85,568
*200 Crosses*										
1	800	11,200	800	11,200	13,600	248,000	200	102,400	200	102,400
2	2,400	35,200	2,400	47,200	2,400	47,200	7,316	113,392	7316	163,392
3	5,280	53,760	5,280	116,160	5,280	116,160	3,840	50,880	3840	50,880
4	3,840	49,280	3,840	53,280	3,840	49,280	2,080	25,600	2080	25,600
5	1,900	23,760	1,900	23,880	1,900	23,280	—	—	—	—
Total	14,220	173,200	14,220	251,720	27,020	483,920	13,436	292,272	13,436	342,272

*Conv*, conventional phenotypic selection breeding program; *GSF1*, genomic selection breeding program, with F_1_ individuals, used as phenotypes in the genomic model calibration; *GSTC*, genomic selection breeding program with parents of the test crosses used in the genomic model calibration; *DH*, doubled haploid breeding program with phenotypic selection; and *DHGS*, doubled haploid breeding program with genomic selection.

### Software implementation

The scenarios previously mentioned were implemented in the R package AlphaSimR (Gaynor *et al*. 2021). We used R (version 4.1.2) for post-processing the outputs from the analyses and the package “ggplot2” (Wickham *et al*. 2016) to generate the graphical outputs. All codes used were made publicly available on GitHub (https://github.com/Resende-Lab/Coelho_Peixoto-Sweet_Corn_DH-GS_simulation).

## Results

### Parental selection and genomic selection implementation

Across the scenarios involving different degrees of parental substitution, several trends were observed, regardless of the program size (50 vs 200 crosses), trait heritability (high vs low), or presence or absence of GE interaction. First, for the *GSF1* scenarios, replacing all the old parents with new parents (0:1 substitution ratio) resulted in the highest genetic gain, except for a single instance; in the 200-cross population, low heritability, and absence of GE, *GSF1* scenario, replacing half of the parents (1:1 substitution ratio) resulting in the highest genetic gain (Tables 2 and3). Likewise, for the *Conv* and *GSTC* scenarios, regardless of all other factors, replacing three-quarters of the parents (1:3 substitution ratio) resulted in the highest genetic gain, except for one scenario; in the 50-cross, low heritability, absence of GE, *GSTC* scenario, substituting half of the parents (1:1 substitution ratio) resulted in the highest genetic gain.

**Table 2.** Genetic mean and average genetic variance for the final generation of parents for simulations with 50 crosses per generation.

Parental parameters	Program	Absence of GE				Presence of GE			
		1:3	1:1	3:1	0:1	1:3	1:1	3:1	0:1
**High heritability**									
Genetic mean	*Conv*	127.8 (2.8)	130.5 (3.9)	131.9 (3.1)	124 (3.9)	114.4 (3.9)	117.4 (4.7)	118.9 (5.5)	112.6 (4.8)
	*GSTC*	124.5 (4.6)	128.2 (4.8)	130.1 (4.4)	122.5 (5.3)	118.5 (4.9)	121 (6.4)	125.7 (5.5)	115.8 (5.5)
	*GSF1*	110.1 (5)	113.3 (5.9)	117.5 (5.6)	127.7 (4.7)	102.2 (5.6)	106.4 (6.6)	110 (6.7)	120.7 (5.4)
Average genetic variance	*Conv*	17.6 (6.3)	15.6 (6.1)	14.6 (4)	9.5 (3)	22 (10.8)	22.4 (22.7)	21.1 (19.3)	9.7 (2.9)
	*GSTC*	23.2 (9.4)	20.3 (7.2)	22.7 (25.1)	10.2 (3.3)	10.5 (4.2)	7.7 (4.3)	7.7 (2.8)	4.2 (1.5)
	*GSF1*	35.6 (13.2)	36 (14.8)	51.6 (20.1)	9.1 (2.3)	31.5 (13)	45 (21.2)	55.8 (26.1)	9 (2.8)
**Low heritability**									
Genetic mean	*Conv*	113.8 (4.1)	117.2 (4)	117.5 (4.3)	112.2 (4.7)	105.3 (4.3)	107.9 (4.4)	108.2 (4.5)	104.6 (4.4)
	*GSTC*	109.8 (5.2)	114.9 (5.7)	114.8 (6.4)	110.3 (5.9)	106.3 (5.7)	107.6 (5.4)	110 (5)	105.5 (5.9)
	*GSF1*	101.7 (5.1)	103.7 (4.9)	106.7 (4.7)	117.1 (4.7)	95.2 (5.5)	98.7 (6.5)	101.1 (6.4)	113.1 (4.9)
Average genetic variance	*Conv*	22.3 (6.4)	21.8 (6.8)	19.3 (5.1)	14.6 (4)	24.5 (7.1)	22.8 (7.2)	20.5 (4.5)	14.6 (4.6)
	*GSTC*	26 (9)	25.2 (7.9)	21.4 (8.3)	13.9 (3.9)	10 (4.8)	9.3 (4.7)	9.1 (4.2)	5.1 (2)
	*GSF1*	35.7 (15)	43.7 (18.9)	51.3 (18.3)	12.8 (3.8)	36.1 (12.2)	44.3 (19.6)	54 (23.9)	13.4 (4.6)

The ratios stand for old parents and new parents selected as candidates to parents at the beginning of each cycle for the crossing block. Numbers in parenthesis refer to the standard error of the estimates.

GE, genotype-by-environment interaction; *Conv*, conventional phenotypic selection scenario; *GSF1*, genomic selection scenario using F_1_ phenotypes to calibrate the genomic model; *GSTC*, genomic selection scenario using the testcross parents’ phenotypes to calibrate the genomic model.

**Table 3.** Genetic mean and average genetic variance for the final generation of parents for simulations with 200 crosses per generation.

Parental parameters	Program	Absence of GE				Presence of GE			
		3:1	1:1	1:3	0:1	3:1	1:1	1:3	0:1
**High heritability**									
Genetic mean	*Conv*	136.2 (3.3)	139.2 (3.0)	140.2 (3.3)	135.6 (3.6)	122.1 (4.4)	125.7 (5.9)	128.2 (4.8)	124.2 (4.4)
	*GSTC*	144.1 (4.2)	150.3 (4.5)	152.4 (4.4)	147.6 (4.9)	138.4 (4.5)	145.4 (5.3)	148.1 (5.3)	142.4 (4.9)
	*GSF1*	129 (4.2)	138.1 (5.8)	144.3 (4.3)	146.6 (4.1)	123.5 (5.7)	131.4 (5.7)	138.8 (4.2)	141.4 (5.4)
Average genetic variance	*Conv*	19 (4.5)	18.3 (5.2)	15.6 (2.9)	11.9 (1.8)	31 (31)	26 (19.9)	20.8 (7)	13.6 (3.0)
	*GSTC*	14.5 (3.9)	12.5 (2.7)	12.1 (2.7)	10 (2)	16 (4.9)	13.7 (4.5)	12.5 (3)	9.7 (2.1)
	*GSF1*	40.7 (17.2)	37 (16.8)	23.9 (6.6)	15 (3)	38.7 (19.1)	40.8 (23.9)	25.7 (11.6)	14.4 (2.3)
**Low heritability**									
Genetic mean	*Conv*	121.5 (3.9)	124.6 (3.4)	125.8 (3.2)	121.9 (3.6)	114 (4.2)	116.4 (4.3)	117.9 (3.7)	114.6 (4.7)
	*GSTC*	129.4 (5.2)	134.5 (5.2)	137.4 (4.6)	132.6 (4.6)	127.5 (5.2)	131.7 (4.9)	133.1 (4.6)	130.6 (5.8)
	*GSF1*	114.3 (4.5)	116.3 (4.2)	117.2 (3.4)	115.3 (3.3)	116.5 (5.3)	125.1 (5.2)	129.3 (6.1)	133.3 (4.4)
Average genetic variance	*Conv*	26.1 (6)	24 (4.5)	22.1 (3.6)	18.3 (3.5)	32.7 (21)	27.4 (7.2)	26.1 (10.5)	19.5 (4.2)
	*GSTC*	16.9 (3.8)	16.8 (4)	15.6 (3.3)	14.4 (3.2)	18.3 (5.4)	16.2 (4.5)	15.1 (3.2)	13.6 (2.8)
	*GSF1*	30.2 (13)	27.6 (19.8)	31.1 (28.9)	18.5 (4.3)	43.4 (16)	38 (17.7)	37.5 (19.4)	19.8 (3.4)

The values represent the mean of 50 repetitions and the standard deviation is reported between parentheses. The ratios stand for the relation in-between the old parents and new parents selected as candidates to parents at the beginning of each cycle for the crossing block. Numbers in parenthesis refer to the standard error of the estimates.

GE, genotype-by-environment interaction; *Conv*, conventional; *GSF1*, genomic selection scenario using F_1_ phenotypes to calibrate the genomic model; *GSTC*, genomic selection scenario using the testcross parents’ phenotypes to calibrate the genomic model.

In terms of genetic variance, the degree of parental substitution that resulted in the highest genetic variance changed between scenarios, without a predominant best performer. However, in every simulated scenario, replacing all parents with new lines (0:1 ratio) resulted in a significant drop in genetic variance after 20 years (Table 2).

As expected, scenarios involving GE interactions exhibited lower genetic means and higher genetic variability after 20 years of breeding when compared to scenarios without GE interaction. For instance, in the scenarios with 50 crosses and low heritability, the performance of scenarios with a GE interaction was outperformed by those scenarios without a GE interaction by 8.12% (*Conv*), 4.75% (*GSTC*), and 5.16% (*GSF1*). Likewise, in the simulations with 200 crosses and a trait with high heritability, the scenarios with a GE interaction surpassed those without a GE interaction by 10.2% (*Conv*), 3.50% (*GSTC*), and 4.26% (*GSF1*).

It is notable that scenarios simulated with traits of higher heritability, on average, achieved more substantial gains at the end of 20 years when compared to traits with lower heritability (*i*.*e*. 50 crosses: 10.2%, 12.1%, and 8.4% higher for *Conv*, *GSTC*, *GSF1* scenarios, respectively; 200 crosses: 9.8%, 10.6%, and 12.9% higher for *Conv*, *GSTC*, *GSF1* scenarios, respectively).

### Breeding strategies simulation

**Fig. 3.**
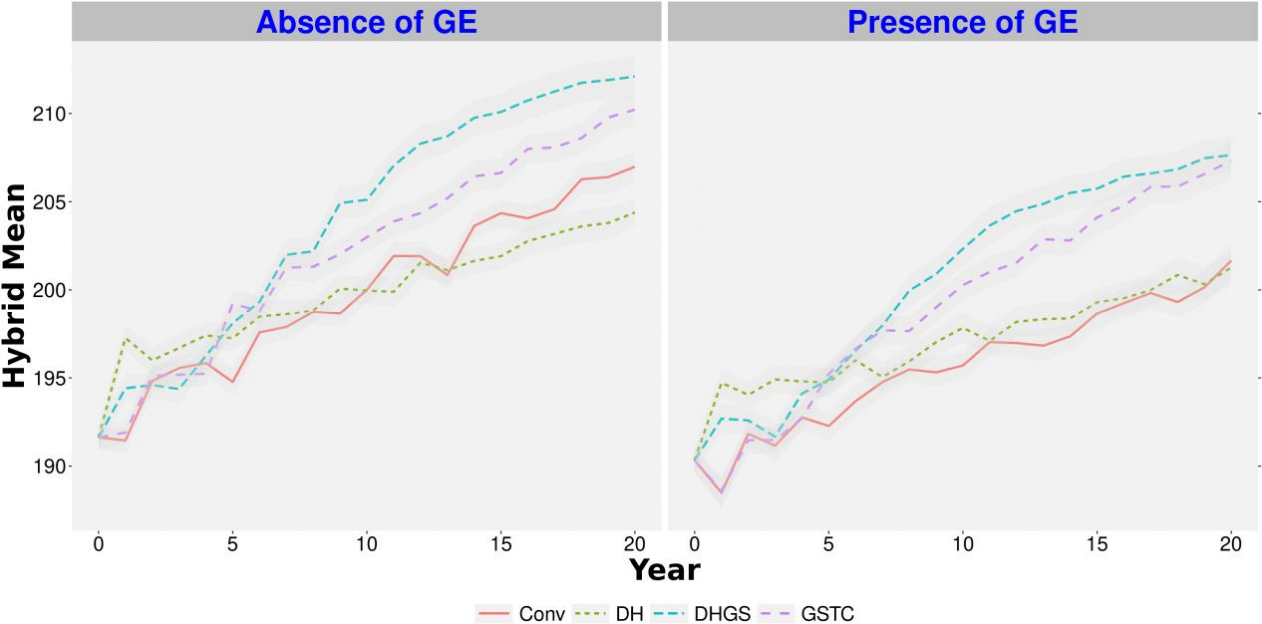
Hybrid genetic mean for a trait with low heritability, in a program with 50 crosses per generation over 20 years of breeding. *Conv*, conventional breeding program; *GSTC*, genomic selection using testcross's parents as phenotypes to calibrate the genomic model; *DH*, doubled haploid scenario; *DHGS*, doubled haploid scenario with genomic selection; GE, genotype-by-environment interaction. The hybrid genetic mean is plotted as a mean of the hybrids for each cycle. Each line represents the hybrid mean over 50 replicates. The standard error of the predictions is represented by the shading around the line for each scenario.

**Fig. 4.**
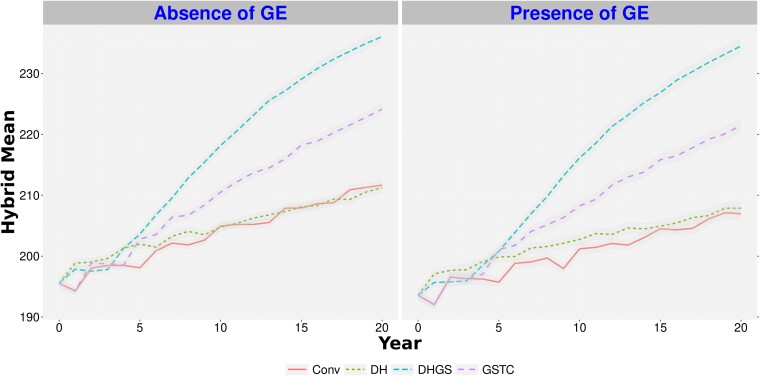
Hybrid genetic means for a trait with low heritability, in a program with 200 crosses per generation over 20 years of breeding. *Conv*, conventional breeding program; *GSTC*, genomic selection using testcross's parents as phenotypes to calibrate the genomic model; *DH*, doubled haploid scenario; *DHGS*, doubled haploid scenario with genomic selection; GE, genotype-by-environment interaction. The hybrid genetic mean is plotted as a mean of the hybrids for each cycle. Each line represents the hybrid mean over 50 replicates. The standard error of the predictions is represented by the shading around the line for each scenario.

The top-performing *Conv* parental substitution ratio, 1:3 old parents to new parents, and the top-performing genomic selection training population model, *GSTC* with 1:3 parental substitution, were chosen for comparison with the *DH* and *DHGS* simulations. Among these comparisons, the hybrid mean after 20 years of selection was lowest for the *Conv* scenario, regardless of all other factors that were considered (Figs. 3 and 4, Supplementary Figs. 1 and 2, and Supplementary Table 2). The performance of the *DHGS* scenario resulted in the highest hybrid mean. The advantage of *DHGS* over *GSTC* for hybrid mean gain was greater in the scenario with 200 crosses (around 5%, Supplementary Fig. 1) than in the scenario with 50 crosses (less than 1%, Supplementary Fig. 2).

**Fig. 5.**
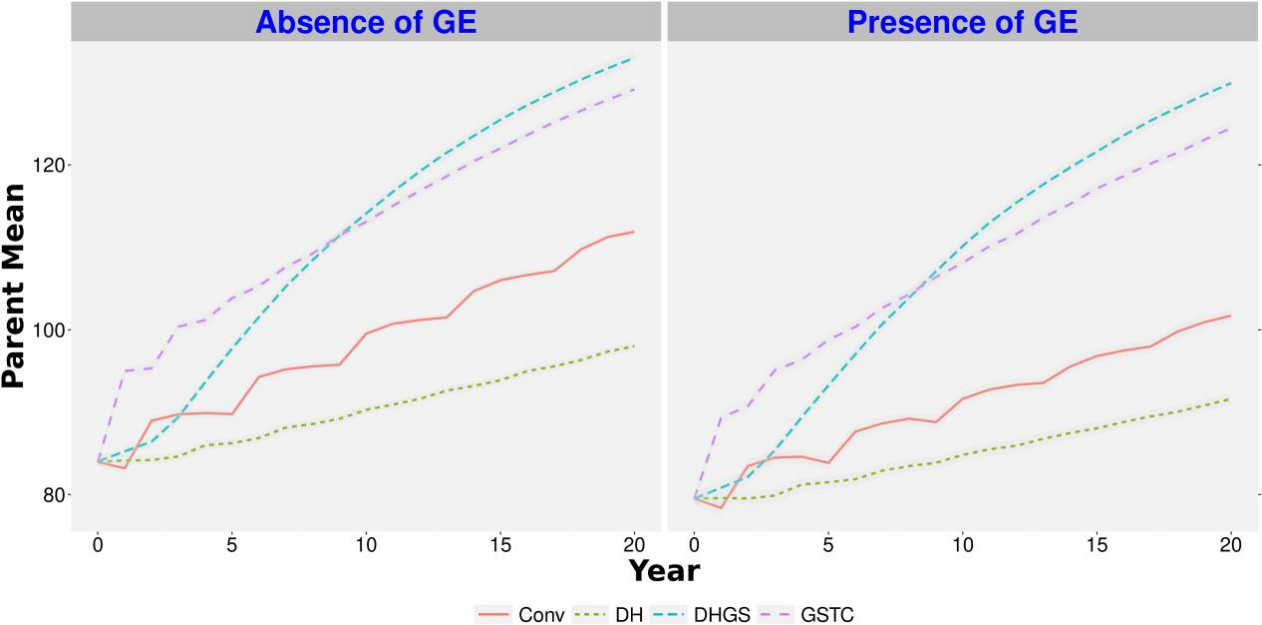
Parental genetic means for a trait with low heritability, in the program with 200 crosses per generation over 20 years of breeding. *Conv*, conventional breeding program; *GSTC*, genomic selection using testcross's parents as phenotypes to calibrate the genomic model; *DH*, doubled haploid scenario; *DHGS*, doubled haploid scenario with genomic selection; GE, genotype-by-environment interaction. The genetic mean is plotted as a mean of the parents for each cycle. Each scenario is represented as the parental genetic mean averaged over 50 replicates. The standard error of the predictions is represented by the shading around the line for each scenario.

**Fig. 6.**
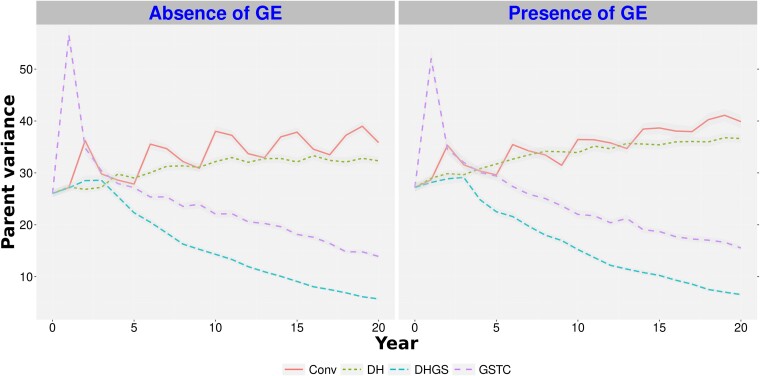
Parental genetic variance trends for a trait with low heritability, in the program with 200 crosses per generation over 20 years of breeding. *Conv*, conventional breeding program; *GSTC*, genomic selection using testcross's parents as phenotypes to calibrate the genomic model; *DH*, doubled haploid scenario; *DHGS*, doubled haploid scenario with genomic selection; GE, genotype-by-environment interaction. The average parental variance is plotted as a mean of the parents’ variance for each cycle. Each scenario is represented as the parental variance averaged over 50 replicates. The standard error of the predictions is represented by the shading around the line for each scenario.

By comparing the parental genetic gain and variances through 20 years, we could see *DHGS* scenario outperformed *GSTC*, no matter the GE scenario or heritability, in the 50 cross-simulations (Supplementary Table 3). Genetic gain in the 200 cross-scenarios demonstrated a different pattern. The *DHGS* had the best genetic gain, surpassing *GSTC*, regardless of trait heritability or GE (Fig. 5), but also demonstrated a steady drop in genetic variance (Fig. 6). In contrast, the *DH* scenario was outperformed by all scenarios, including the *Conv* scenario, regarding genetic gain (Fig. 5), but it maintained a steady positive genetic variance trend (Fig. 6). In addition, the number of years to complete the whole breeding cycle (from the generation of crosses to the release of the lines) decreased from 5 years (*Conv* and *GSTC* scenarios) to 4 years (*DH* and *DHGS* scenarios). This had an impact on the number of released material throughout the 20 years of breeding simulation, which is supposed to be eight lines released for *Conv*/*GSTC* (four full cycles in 20 years) and 10 lines released for *DH*/*DHGS* (five full cycles in 20 years).

The *Conv* budget (Table 4) mimics the current approximated costs of a small, sweet corn breeding program. The calculated budget included costs of evaluation and pollination, applied technologies, and personnel. Overall, the implementation of genomic selection technology increases the costs considerably from *Conv* to *GSTC* and from *DH* to *DHGS*, mainly because of the associated genotyping costs. For the latter two, in addition to genotyping costs, the production of doubled haploid increased costs. *DHGS* had the highest budget (Table 4), around two times more expensive than *Conv*, even though the number of field plots was smaller. Scenarios with doubled haploids (*DH* and *DHGS*) demonstrated a lower efficiency than the *Conv* and *GSTC*. Yet these methods (*DH* and *DHGS*) achieved the highest hybrid gain, for both trait heritabilities and both program sizes. GE interaction decreased the cost efficiency.

**Table 4.** Cost efficiency of breeding strategies based on size/number of plots, time in years, and performance based on hybrid gain and costs.

Scenario	# of Years	Total Plots	Annual Cost ($)	Relative Gain*^a^*		Cost efficiency*^b^*	
				No GE	GE	No GE	GE
**50 crosses**							
*Low heritability*							
*Conv*	5	3,555	46,780	1.00	1.00	1.00	1.00
*GSTC*	5	3,555	62,930	0.99	1.00	1.36	1.35
*DH*	4	3,359	73,068	1.02	1.03	1.52	1.52
*DHGS*	4	3,359	85,568	1.02	1.03	1.80	1.78
*High heritability*							
*Conv*	5	3,555	46,780	1.00	1.00	1.00	1.00
*GSTC*	5	3,555	62,930	0.99	1.04	1.29	1.29
*DH*	4	3,359	73,068	1.03	1.00	1.56	1.56
*DHGS*	4	3,359	85,568	1.02	1.05	1.75	1.75
**200 crosses**							
*Low heritability*							
*Conv*	5	14,220	173,200	1.00	1.00	1.00	1.00
*GSTC*	5	14,220	251,720	1.06	1.07	1.37	1.36
*DH*	4	13,436	292,272	1.00	1.00	1.69	1.68
*DHGS*	4	13,436	342,272	1.12	1.13	1.77	1.74
*High heritability*							
*Conv*	5	14,220	173,200	1.00	1.00	1.00	1.00
*GSTC*	5	14,220	251,720	1.07	1.09	1.36	1.34
*DH*	4	13,436	292,272	1.00	1.01	1.68	1.66
*DHGS*	4	13,436	342,272	1.12	1.14	1.76	1.73

The relative gain is measured in the hybrid component. *Conv*, conventional breeding program; *GSTC*, genomic selection using testcross's parents as phenotypes to calibrate the genomic model; *DH*, doubled haploid scenario. *DHGS*, doubled haploid scenario with genomic selection; GE, genotype-by-environment interaction. # of years represents the number of years to complete a cycle of breeding (from crosses to lines release).

^*a*^The relative gain assumes the hybrid gain of the *Conv* scenario as a unit (1) and scales the other scenarios’ hybrid gain for the *Conv* scenario.

^*b*^The cost efficiency was measured by dividing the relative gain by the relative costs (assuming the costs of the *Conv* scenario as a unit and scaling the other scenarios’ costs to that one).

## Discussion

In our simulation study, we explored the best strategies to update the parental population at the beginning of each cycle and strategies to implement genomic selection and doubled haploid technologies, in a recurrent selection program. In addition, we measured their impact on genetic gain and diversity trends, the costs of the implementation of each one, and their influence on breeding and market development for a sweet corn breeding program.

### Parental selection and genomic selection scenarios

Selecting individuals to compose crossing blocks at the start of each breeding cycle is crucial in breeding programs. Replacing all individuals each cycle, while beneficial for phenotypic-based selection (Labroo and Rutkoski 2022), did not prove optimal in our simulations, even for traits with lower heritability affected by GE interaction. Labroo and Rutkoski argued that replacing all parents reduces selection error bias, which can inflate genetic values. This strategy prevents selected individuals from being carried over multiple cycles. Additionally, for genomic selection, discrete and overlapping strategies show similar performance (Labroo and Rutkoski 2022). The selection bias in genomic models is lower due to more accurate estimated breeding values compared to phenotypic selections.

However, our results showed significant long-term impacts, such as reduced genetic diversity and smaller genetic gains, when all individuals were replaced (*Conv* scenario). A 1:3 replacement ratio was found ideal for all scenarios, including genomic selection. In our simulations, only parents from the last cycle and new parent candidates were included in the crossing block, unlike in Labroo and Rutkoski's simulations where all individuals were retained throughout cycles. Replacing all individuals every few cycles can propagate selection error bias, thus supporting the use of an overlapping strategy (1:3 ratio) over the discrete strategy. Under our assumptions, mixing new lines with a small proportion of old individuals appears optimal for both genomic and phenotypic selection.

As demonstrated by the *GSTC* and *GSF1* scenarios, using the phenotypes of the testcrosses’ parents was the best approach to deploy genomic selection in the sweet corn breeding program. Especially for traits with small heritability, measuring phenotypes in advanced/later trials, such as testcross, in the target environment, and across a large set of trials, lead to a better estimation of marker effects (Berro *et al*. 2019; Verges and van Sanford 2020). Thus, all these factors could have contributed to the improvement of the model accuracy, and, together with a lower number of individuals to be genotyped compared with the *GSF1* scenario, demonstrated that the *GSTC* is ideal for implementation.

### Genotype-by-environment interaction and the decay in genetic variation

When determining the most efficient strategy to be implemented in a breeding program and its impact on the trends of genetic mean and variance, it is important to include a reference scenario that lacks a GE interaction (Cowling *et al*. 2020; Bančič *et al*. 2021). However, GE interaction plays an important role in real breeding programs, and methodologies accounting for GE interaction have been widely applied (Crossa *et al*. 2006, 2017; Heslot *et al*. 2012; Jarquín *et al*. 2016). In the presence and absence of a GE interaction, the *DHGS* strategy performed best. It is well known that GE interaction brings noise to the selection process and may largely impact it, especially phenotypic selection. Therefore, scenarios simulated with genomic selection (*GSTC* and *DHGS*) presented a higher performance (being the *DHGS* the most efficient) compared with those without genomic selection (*Conv* and *DH*). These results suggest that genomic selection may boost the selection of better materials, once the selection is based on their estimated genetic value, which is less impacted by GE interaction, instead of their phenotypic value, which is highly impacted by GE interaction. It is worth highlighting that the genomic selection pipelines allowed to performance of off-season selection, which is an advantage compared with phenotypic-base scenarios (*DH* and *Conv*). This can aid in overcoming the GE interaction's impact on the selection of better genotypes in the sweet corn program.

It is important to emphasize that, within the context of the *DHGS* scenario, there was a large decline in genetic variation. This poses a critical challenge for breeding programs since a lack of genetic diversity hampers the long-term potential for genetic improvement. To balance between the combined advantage of doubled haploid with genomic selection and the loss of genetic diversity, two possible solutions are to utilize either optimum contribution selection (Meuwissen and Sonesson 1998) or optimal cross selection (Gorjanc *et al*. 2018). Optimum contribution selection aims to maximize target criteria by optimizing parental contributions in a series of crosses while simultaneously minimizing inbreeding rates in the subsequent generation (as a proxy for genetic variation). This strategy preserves genetic diversity, thereby ensuring the sustainability of the breeding program. Similarly, optimal cross selection involves identifying a group of crosses that maximizes the specified criteria and minimizes inbreeding rates in the subsequent generation. Both techniques have been recognized as crucial tools for managing genetic diversity and enhancing genetic gains, making them valuable assets in the realm of plant breeding programs (Allier *et al*. 2019; Obšteter *et al*. 2019; Labroo and Rutkoski 2022; Pocrnic *et al*. 2023). The combined use of doubled haploids with either optimum contribution or optimal cross selection methods has the potential to not only bolster genetic gains but also effectively address the decline in genetic variance, offering a promising strategy to be further explored in the sweet corn program.

### Combining genomic selection and doubled haploid technology

Even though the *Conv* and *GSTC* strategies had lower costs per year, they had to employ successive seasons of self-pollination and selection until genotypes could be crossed with testers to evaluate the GCA with elite lines. In this way, the implementation of doubled haploid technology brought a reduction in the amount of self-pollination, reducing labor and shortening the breeding program length from 5 to 4 years. However, both doubled haploid strategies (*DH* and *DHGS*) increased costs by at least 56% and 80% compared to the *Conv* strategy (*DH* and *DHGS*, respectively). Several studies have reported the superiority of genomic selection over a conventional pipeline (Gaynor *et al*. 2017; Muleta *et al*. 2019; Bančič *et al*. 2021; Sabadin *et al*. 2022; Peixoto *et al*. 2023), and we observed a similar pattern in this study, regardless the presence or absence of GE and the trait heritabilities. Generally, the application of only genomic selection shortens the breeding cycle (Fritsche-Neto *et al*. 2021; Volpato *et al*. 2021). However, in the sweet corn breeding program that we simulated, shortening the cycle by implementing genomic selection is not feasible. Moreover, the focus of genomic selection is related to increasing selection accuracy in the off-season environment selections. In our case (same cycle length), genomic selection represents the optimum strategy to be applied (higher hybrid gain). In addition, genomic selection was more cost-efficient than *DHGS* under the same simulated conditions. It is worth mentioning that in many hybrid crops, such as sweet corn, the use of genomic selection can be used to predict specific hybrid combinations as a way to decide which ones represent the most promising combinations (Schrag *et al*. 2019; Zystro *et al*. 2021a; Peixoto *et al*. 2024).

It is important to note that while the cost efficiency for *DH* and *DHGS* is lower under simulated GE conditions, the total investment under these simulated settings is also higher. Hence, it is more expensive to deploy these technologies than it is to carry out a conventional program. However, it is worth noting that as large as the breeding program, even more hybrid gain could be harnessed, as highlighted by the trends found in the *DHGS* scenario. This is relevant, particularly in public or small-scale breeding programs where total budgets may be limited. These results, however, are tied to our estimated cost for each technology, which may not be accurate depending on the genotyping platform, field costs, and availability of internally doubled haploid services. We expect that breeding simulations could have a big impact in enabling breeding programs to customize their breeding programs to deploy technologies that can increase the genetic gain and cost efficiency, while still fitting in their overall annual budget.

Because of the 4-year cycle, instead of the 5-year cycle as in *Conv* scenario, in 20 years of breeding the *DH* and *DHGS* strategies released 20% more lines than the *Conv* scenario with higher gains. Thus, doubled haploids positively impact decreasing the time-to-market and increasing the market share (*i*.*e*. the number of varieties released). In addition, *DHGS* as discussed, was even more efficient under the presence of GE interactions. Thus, if we consider the successful development of the spontaneous doubling in the next few years for sweet corn, the efficiency can increase even more, where the development and maintenance of the homozygous line of the program would be facilitated. The number of self-pollination, which is very laborious, would decrease dramatically after the first year (generation of doubled haploid and seed increase). Also, in terms of market share, the competitiveness of a breeding program through the generation of new hybrids and lines will increase, helping to generate revenue for the program which can be reinvested to further improve these techniques.

### Assumptions and further implications for the sweet corn breeding program

Even though we explored traits with different genetic controls and the impact of GE interaction, the scenarios here simulated only targeted one trait for selection and improvement at a time. However, sweet corn breeders have historically selected for multiple traits (Zystro *et al*. 2021b; Neto *et al*. 2022; Peixoto *et al*. 2024) which is known to decrease the overall performance of the genetic gains for the traits, for both, phenotypic- and genomic-based selections (Peixoto *et al*. 2021; Silva, Peixoto, *et al*. 2021). To extrapolate these scenarios to a multitrait framework, a selection index could be implemented with weights assigned for the simultaneous selection of all traits. This would likely impact the performance of the scenarios explored in this study, changing the applicability and feasibility of these approaches to a sweet corn breeding program.

To mimic the sweet corn breeding program pipeline at the UF, two pseudo-heterotic groups were simulated. However, the different breeding strategies were only simulated to affect the UF pseudo-heterotic group, effectively ignoring the other pseudo-heterotic group. Reciprocal recurrent selection into the sweet breeding program with two pools of breeding (such as heterotic groups) can lead to different performances of the scenarios *DH* and *DHGS*, and even increase the genetic gain (de Jong *et al*. 2023). Based on this, we hypothesize that applying doubled haploids for both pseudo-heterotic groups could improve even more the benefits of the *DHGS* program. However, further investigation needs to be done.

This study investigated the potential of implementing new breeding tools into the sweet corn breeding program using simulation. We demonstrated that these tools could be used to increase the genetic gain of hybrids generated in partnership with private companies and of the parental lines. There was an associated increase in cost, which can be a difficult threshold for public programs to overcome, as they are often limited by physical space and project funding. However, the investments brought real gains to genetic means and a reduction in time to achieve those gains. Over time, the cost of these tools may be offset by increasing market share and increased recognition as an institution that generates good elite inbreeds and, consequently, better hybrids faster. Also, the new technologies can help to decrease the opportunity cost associated with making large numbers of self-pollinations and selections every season to advance the population.

Some of the challenges encountered during this analysis could be further investigated but were not the focus of this work. The genomic model developed for implementation in genomic selection could be further improved by investigating the use of different models such as semiparametric, nonlinear, or Bayesian models, examining the effects of using a historical empirical dataset as the training population, and/or, applying the genomic selection at a different stage (*e*.*g*. hybrid prediction in the TCs or for cross prediction and mate pair allocation). However, in this study, AlphaSimR was both reliable and robust in exploring breeding scenarios involving genomic models and doubled haploid populations. Additionally, while this study focused on estimating and comparing the explicit costs associated with these tools, a robust cost-benefit analysis could help to better demonstrate the cumulative benefits and identify break-even points to better guide implementation decisions.

## Conclusion

Replacing three-quarters of the parental population with new lines each generation was determined to be the best option to increase the genetic mean in the sweet corn breeding program. Additionally, the utilization of doubled haploids combined with a genomic selection scenario had superior logistical potential, decreasing the number of years to complete a breeding cycle, and increasing the gains per unit of time. The cost analysis reinforced what many breeders are aware of: while genetic improvement is the core of plant breeding it is far from the only factor that must be considered when improving the efficiency of the breeding pipeline. Any decision must consider many tangible and intangible factors, such as the sustainability of genetic gain, the logistics of the process, financial budgets, and the market goals of the breeding program.

## Supplementary Material

jkae128_Supplementary_Data

## Data Availability

The data underlying this article are available in the GitHub repository (https://github.com/Resende-Lab/Coelho_Peixoto-Sweet_Corn_DH-GS_simulation). Supplemental material available at G3 online.
