# Supplementary material for: Use of simulation to optimize a sweet corn breeding program: implementing genomic selection and doubled haploid technology
Source: G3 (Bethesda). 2024 Jun 13;14(8):jkae128. doi: 10.1093/g3journal/jkae128 (PMC11304600; doi:10.1093/g3journal/jkae128)
Supplement: jkae128_Supplementary_Data [file jkae128_supplementary_data.docx]

**Optimizing a sweet corn breeding program: implementing genomic selection and doubled haploid technology**

Marco Antônio Peixoto^1,2,‡^, Igor Ferreira Coelho^1,2,‡^, Kristen A. Leach^2^, Thomas Lübberstedt^3^, Leonardo Lopes Bhering^1^, Márcio F. R. Resende Jr.^2,*^

^1^ Laboratório de Biometria, Universidade Federal de Viçosa, Viçosa, Minas Gerais, Brazil.

^2^ Sweet Corn Breeding and Genomics Lab, University of Florida, Florida, United States

^3^ Department of Agronomy, Iowa State University, Ames, Iowa, United States

*Corresponding author: Sweet Corn Breeding and Genomics Lab, University of Florida, Florida, United States. Email: mresende@ufl.edu

^‡^ The authors contributed equally to the work


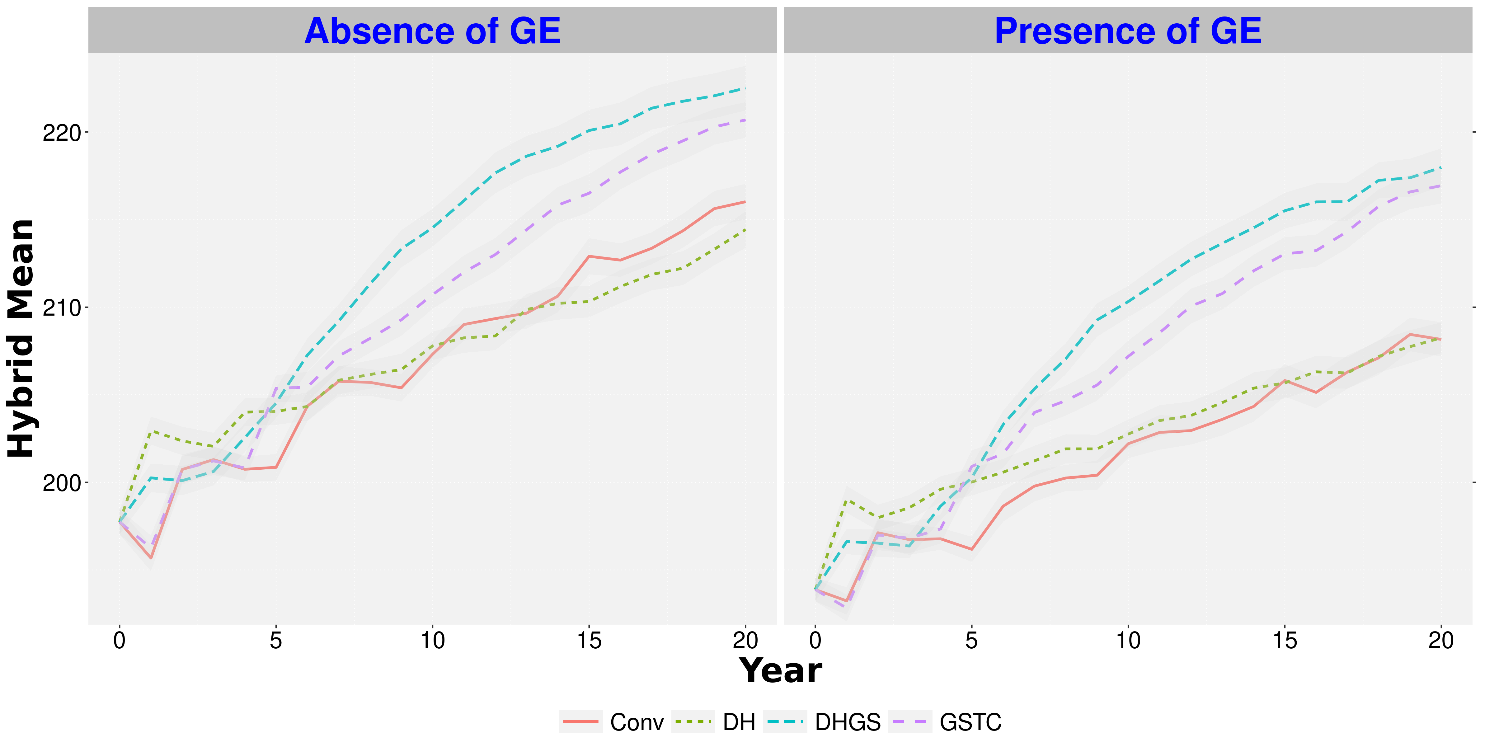
**Figure S1**. **Hybrid genetic mean over 20 years of a breeding program, in the presence and absence of genotype-by-interaction effect (G×E) for a trait with higher heritability in the program with 50 crosses.** The conventional (*Conv*) and conventional using genomic selection (*GSTC*) strategies were compared to the doubled haploid strategy and doubled haploids with genomic selection (*DH* and *DHGS*, respectively). The lines represent the mean for 50 replicates. The shaded area around the lines represents the standard error for the replicates.


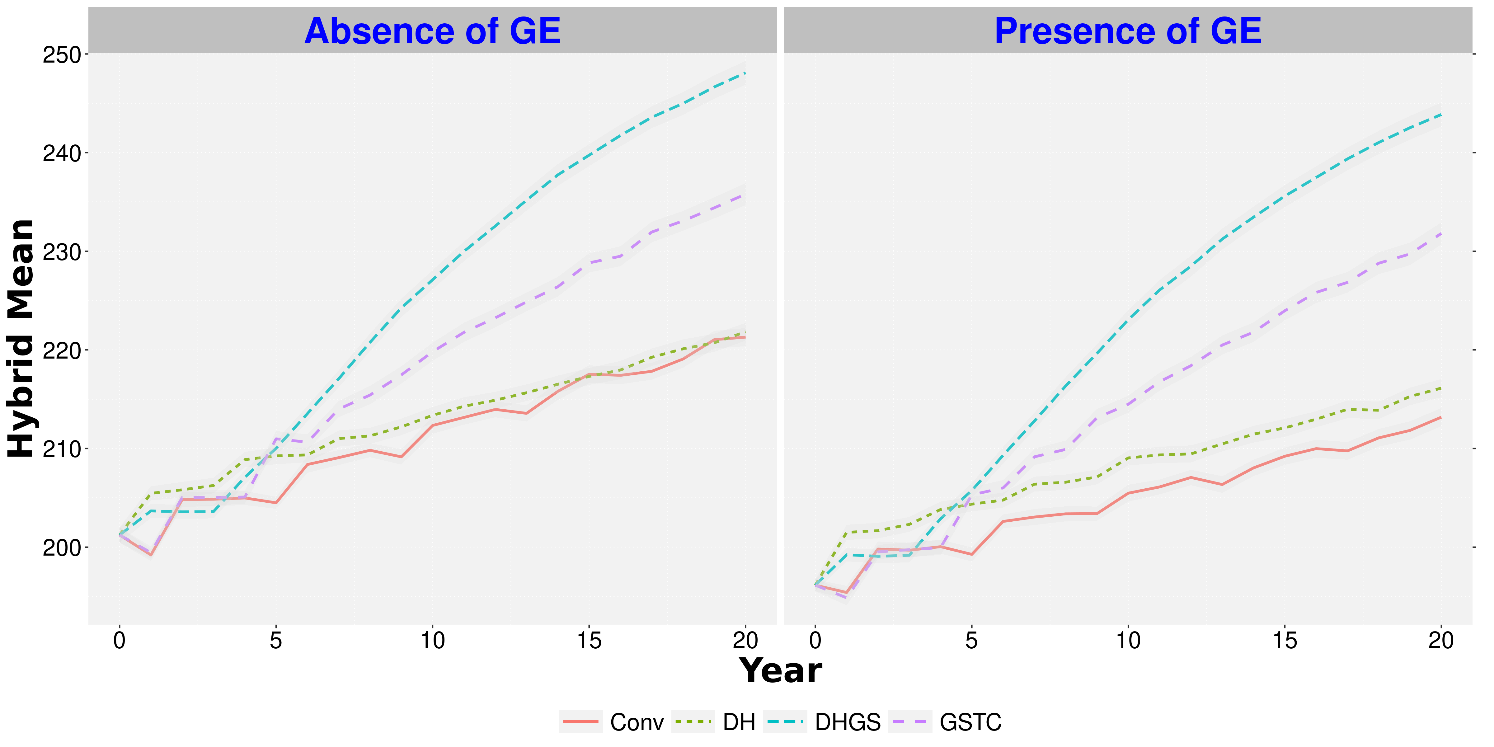


**Figure S2**. **Hybrid genetic mean over 20 years of a breeding program, in the presence and absence of genotype-by-interaction effect (GE) for a trait with higher heritability in the program with 200 crosses.** The conventional (*Conv*) and conventional using genomic selection (*GSTC*) strategies were compared to the doubled haploid strategy and doubled haploids with genomic selection (*DH* and *DHGS*, respectively). The lines represent the mean for 50 replicates. The shaded area around the lines represents the standard error for the replicates.

**Table S1** – Simulation pipeline by phase and stage detailing parameters and features for each.

| Simulation Phases | Breeding Stages | Parameters and Features Information |
| --- | --- | --- |
| Genome sequence | Base Genome | Maize historical effective population size |
|  |  | 10 chromosome pairs (2.0 Morgan each) |
|  |  | 300 QTN/chromosome pairs |
|  |  | Physical length 2×10^8^ base pairs |
|  |  | Mutation rate of 2.5×10^-8^ |
|  |  | Recombination rate of 1.25×10^-8^ |
| Burn-In Years Breeding | Founder Population | 200 inbred lines |
|  |  | 3000 SNP/chromosome |
|  |  | QTN effects under normal distribution |
|  | Past Years Breeding | ADG trait |
|  |  | 20 years of baseline breeding program |
| Future Years Breeding | Future Years Breeding | ADG trait |
|  |  | Test two GE and two heritability levels |
|  |  | Implement different pipelines |
|  |  | Compare strategies |
|  |  | Calculate hybrid gains |
|  |  | Compare strategies’ costs and effectiveness |

*ADG, a trait controlled by additive, dominance, and the genotype-by-environment (GE) interaction effects; SNP, single nucleotide polymorphism; QTN, quantitative trait nucleotide.

**Table S2** – **Hybrid genetic mean at year 20 of the simulated breeding programs, in the presence and absence of genotype-by-interaction effect (GE) for traits with lower and high heritability in the program with 50 and 200 crosses.** The conventional (*Conv*) and conventional using genomic selection (*GSTC*) strategies were compared to the doubled haploid strategy and doubled haploids with genomic selection (*DH* and *DHGS*, respectively).

| **Scenario** | **GE** | **High heritability** | | | **Low heritability** | | |  |
| --- | --- | --- | --- | --- | --- | --- | --- | --- |
|  |  | **Hybrid Mean** | **%** | | **Hybrid Mean** | **%** | |  |
| **50 Crosses** | | | | | | | |  |
| ***Conv*** | No GE | 216 | | 0 | 206.9 | | 0 |  |
| ***GSTC*** | No GE | 220.6 | | 0.7 | 210.2 | | 1.5 |  |
| ***DH*** | No GE | 214.4 | | 3 | 204.3 | | -1.2 |  |
| ***DHGS*** | No GE | 222.5 | | 2.1 | 212.1 | | 2.4 |  |
| ***Conv*** | With GE | 208.1 | | 0 | 201.6 | | 0 |  |
| ***GSTC*** | With GE | 216.9 | | 4.2 | 207.3 | | 2.8 |  |
| ***DH*** | With GE | 208.2 | | 0.1 | 201.2 | | -0.2 |  |
| ***DHGS*** | With GE | 217.9 | | 4.7 | 207.6 | | 2.9 |  |
| **200 Crosses** | | | | | | | |  |
| ***Conv*** | No GE | 213.1 | | 0 | 206.9 | | 0 |  |
| ***GSTC*** | No GE | 231.8 | | 8.7 | 221.5 | | 7 |  |
| ***DH*** | No GE | 216.1 | | 1.3 | 207.8 | | 0.4 |  |
| ***DHGS*** | No GE | 243.8 | | 14.3 | 234.5 | | 13.3 |  |
| ***Conv*** | With GE | 221.2 | | 0 | 211.6 | | 0 |  |
| ***GSTC*** | With GE | 235.7 | | 6.5 | 224.1 | | 5.8 |  |
| ***DH*** | With GE | 221.8 | | 0.2 | 211.2 | | -0.2 |  |
| ***DHGS*** | With GE | 248 | | 12.1 | 236 | | 11.5 |  |

**Table S3** – **Parental genetic mean and variance at year 20 of the simulated breeding programs, in the presence and absence of genotype-by-interaction effect (GE) for traits with lower and high heritability in the program with 50 and 200 crosses.** The conventional (*Conv*) and conventional using genomic selection (*GSTC*) strategies were compared to the doubled haploid strategy and doubled haploids with genomic selection (*DH* and *DHGS*, respectively). Numbers in parenthesis refer to the standard error of the estimates.

| **Parental parameters** | | **Absence of GE** | | | |  | **Presence of GE** | | | |
| --- | --- | --- | --- | --- | --- | --- | --- | --- | --- | --- |
|  |  | ***Conv*** | ***GSTC*** | ***DH*** | ***DHGS*** |  | ***Conv*** | ***GSTC*** | ***DH*** | ***DHGS*** |
| **50 crosses** | | |  |  |  |  |  |  |  |  |
| **High heritability** | | | | | | | | | | |
| Genetic mean | | 121.9 (0.4) | 124.5 (0.6) | 106.3 (0.5) | 117.3 (0.8) |  | 106.7 (0.5) | 118.2 (0.6) | 95.2 (0.6) | 110.1 (1) |
| Genetic variance | | 20.4  (1) | 6.2 (0.4) | 18 (0.8) | 0.5 (0.1) |  | 25.3 (1.3) | 5.9 (0.3) | 21.4 (0.8) | 0.6 (0.1) |
| **Low heritability** | | | | | | | | | | |
| Genetic mean | | 106.3 (0.4) | 109.1 (0.6) | 86 (0.7) | 103.8 (0.8) |  | 96.8 (0.5) | 105.6 (0.6) | 86 (0.7) | 98.6 (1.1) |
| Genetic variance | | 33.7 (1.3) | 7.6 (0.5) | 27.6 (1) | 0.9 (0.1) |  | 33.5 (1.5) | 7.6 (0.6) | 28.3 (1.2) | 0.9 (0.1) |
| **200 crosses** | | |  |  |  |  |  |  |  |  |
| **High heritability** | | | | | | | | | | |
| Genetic mean | | 128.5 (0.3) | 145.8 (0.5) | 113.3 (0.4) | 147.5 (0.8) |  | 112.5 (0.5) | 138.5 (0.5) | 102.8 (0.6) | 141.5 (0.7) |
| Genetic variance | | 23.4 (0.5) | 10.9 (0.3) | 22 (0.5) | 5.2 (0.2) |  | 29.1 (0.9) | 11.8 (0.3) | 27.7 (0.7) | 5.1 (0.2) |
| **Low heritability** | | | | | | | | | | |
| Genetic mean | | 111.8 (0.4) | 129.1 (0.6) | 98 (0.4) | 133  (0.8) |  | 101.7 (0.7) | 124.5 (0.7) | 91.6 (0.6) | 129.1 (0.6) |
| Genetic variance | | 35.8 (0.8) | 13.8 (0.5) | 32.3 (0.7) | 5.7 (0.2) |  | 39.8 (1.2) | 15.5 (0.5) | 36.6 (0.8) | 13.8 (0.5) |
